# Supplementary material for: Resolving the full spectrum of human genome variation using Linked-Reads
Source: Genome Res. 2019 Apr;29(4):635–45. doi: 10.1101/gr.234443.118 (PMC6442396; doi:10.1101/gr.234443.118)
Supplement: Supplemental Material [file supp_gr.234443.118_Supplemental_longranger-2.2.zip › longranger-2.2.tar.bz2!/lr2.2-public/lib/stan/cmdstan/stan/doc/api/html/calculate__chain_8hpp_source.html]

Stan: src/stan/agrad/rev/calculate\_chain.hpp Source File


|  |  |
| --- | --- |
|  | Stan  2.5.0  probability, sampling & optimization |


- Main Page
- Related Pages
- Namespaces
- Classes
- Files

- File List
- File Members

All Classes Namespaces Files Functions Variables Typedefs Enumerations Enumerator Friends Macros Pages

- src
- stan
- agrad
- rev

calculate\_chain.hpp

Go to the documentation of this file.

1 #ifndef STAN\_\_AGRAD\_\_REV\_\_CALCULATE\_CHAIN\_HPP

2 #define STAN\_\_AGRAD\_\_REV\_\_CALCULATE\_CHAIN\_HPP

3

4 #include <valarray>

5

6 namespace stan {

7  namespace agrad {

8  inline double calculate\_chain(const double& x, const double& val) {

9  return std::exp(x - val); // works out to inv\_logit(x)

10  }

11  }

12 }

13 #endif

stan::agrad::calculate\_chain

double calculate\_chain(const double &x, const double &val)

**Definition:** calculate\_chain.hpp:8

stan::agrad::exp

fvar< T > exp(const fvar< T > &x)

**Definition:** exp.hpp:16


---

[ Stan Home Page ]

*© 2011–2014,
Stan Development Team.*
